# Supplementary figures and images for: Relationship between insulin resistance surrogate markers with diabetes and dyslipidemia: A Bayesian network analysis of Korean adults
Source: PLoS One. 2025 May 8;20(5):e0323329. doi: 10.1371/journal.pone.0323329 (PMC12061414; doi:10.1371/journal.pone.0323329)

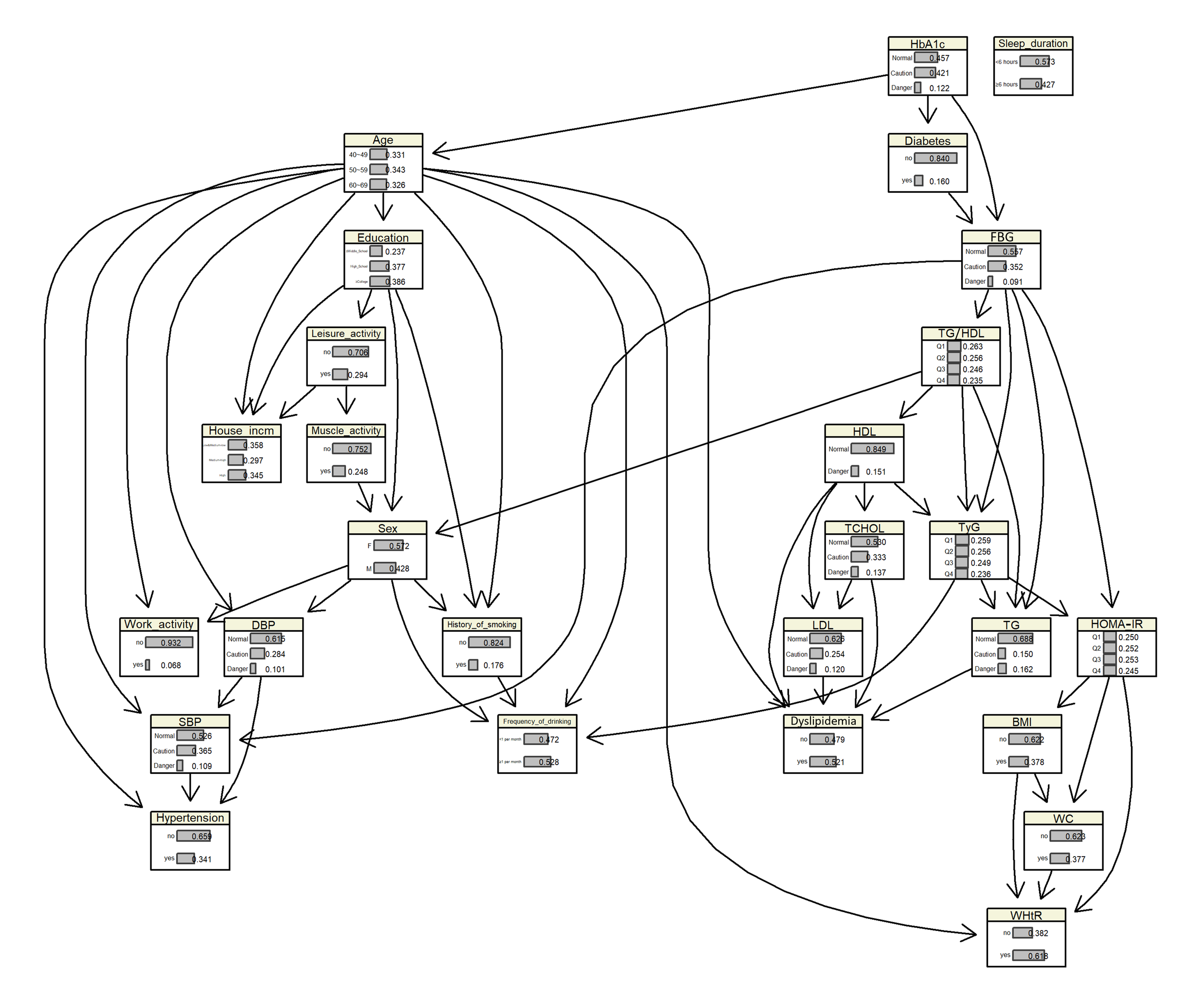

Supplement: S1 Fig — Abbreviation: BMI, body mass index; WC, waist circumference; WHtR, waist-to-height ratio; SBP, systolic blood pressure; FBG, fasting blood glucose; HbA1c, hemoglobin A1c; TG, triglycerides; HDL, high-density lipoprotein; LDL, low-density lipoprotein; TCHOL, total cholesterol; HOMA-IR, homeostasis model assessment of insulin resistance; TyG index, triglyceride-glucose index; TG/HDL ratio, triglyceride-to-high-density lipoprotein cholesterol ratio. (TIF) [file pone.0323329.s001.tif]
