# Supplementary material for: Relationship between insulin resistance surrogate markers with diabetes and dyslipidemia: A Bayesian network analysis of Korean adults
Source: PLoS One. 2025 May 8;20(5):e0323329. doi: 10.1371/journal.pone.0323329 (PMC12061414; doi:10.1371/journal.pone.0323329)
Supplement: Attachment — Submitted filename: Reviewed by Seyedeh Elaheh Bagheri.pdf [file pone.0323329.s002.pdf]

Manuscript Number: **PONE-D-25-06103** reviewed by **Seyedeh Elaheh Bagheri**

## Overall

I read with great interest the article entitled "Relationship between insulin resistance surrogate markers with diabetes and dyslipidemia: A Bayesian network analysis of Korean adults", which falls within the aim of this journal. This study aimed to evaluate the interrelationships among insulin resistance markers (HOMA-IR, TyG index, and TG/HDL ratio) and their associations with diabetes and dyslipidemia using Bayesian network analysis.

In my honest opinion, the topic and results are interesting for audiences and the paper is almost well structured enough to attract the readers' attention. However, the authors should consider and clarify some points and improve the paper, as suggested below [*: major points, #: minor points].

## Abstract section

# Please note that you have considered fasting blood glucose; therefore, it is preferable to use FBG instead of GLU, which is merely an abbreviation for glucose, throughout the text.

# Preferably, replace 'a distinct serial connection pattern' with "a sequential association pattern". Additionally, use 'established association with', instead of "inherent incorporation of". Moreover, instead of "exceptional" in "exceptional tool", which is not a commonly used term in articles, use like 'effective'.

## Material and methods section

\# Preferably, provide a brief explanation alongside the phrase “participants outside the age range of 40–70 years” to clarify the reason for this selection. For example, you did not consider individuals outside this age range because the risk of diabetes and dyslipidemia is not high in those groups.

* In the Study Design and Data Extraction part, you have referenced [27,28] as well as [28] separately. Could you please clarify exactly what these references pertain to and the purpose of citing them?

\# Preferably, include a mention of the participants’ fasting status wherever you discuss the measurement of indices.

\# Preferably, cite the relevant references after the formulas for the TyG index and TG/HDL ratio.

* Please provide some details on how household income was categorized and cite an appropriate reference(s) for it. Additionally, include suitable references for the categorization of sleep and smoking status.

* In the Statistical Analysis part, please specify the comparisons made between the two groups. Additionally, include an explanation of the significance threshold, clarifying the interpretation of P-value < 0.05.

\# Preferably, provide a brief explanation in the text regarding why Rao-Scott chi-square tests were used, instead of Pearson’s Chi-Square Test.

* Since you mentioned that you compared the accuracy of the three markers (HOMA-IR, TyG index, and TG/HDL ratio), please ensure that the interpretations in the following section explicitly incorporate the term accuracy.

**Results section**

* Please ensure that throughout the text and in all tables, the number is presented alongside the percentage, following the format n (%).

* Instead of directly referring to $Q_1$ to $Q_4$, please provide an interpretation of their meaning. Additionally, preferably include an interpretation of their statistical significance. I think that it should be sufficient to mention which group significantly had the highest quartile (Q).

* Before interpreting the structure of Directed Acyclic Graph (DAG) in this section, please first clarify the purpose of using that in the previous section.

* Additionally, it seems likely that you drew the DAG manually rather than using the online DAGitty version 3.1 software. Preferably, recreate the relationships using the software and upload the updated image. This will allow you to identify and correct any cyclic models by adding or removing specific relationships. Moreover, some relationships currently appear incorrectly drawn. For example, education does not influence gender; rather, the reverse is true. Similarly, the relationships between muscle activity and TG/HDL with gender seem misrepresented. Please review and adjust these accordingly.

* Preferably, provide an interpretation of the Markov blanket in Figure 4, making it as clear and intuitive as possible to ensure easier understanding for readers with limited familiarity with the concept. Additionally, briefly mention the purpose of using the Markov blanket in the previous section to provide better context.

* Regarding the sentence “Hypertension was not part of the Markov blanket of any of the three indices; however, it showed the strongest association with the TG/HDL ratio”, please clarify the second part of the sentence. Currently, in Figure 3, there is no line indicating a relationship between them, so I personally do not understand the association between hypertension and the TG/HDL ratio.

**Good *Luck***,

**Confidential comments to the editor**:

Dear Editor-in-Chief: **PLOS ONE**

In my opinion, the paper is probable to be accepted if all points are employed.

Thank you in advance for your invaluable time and consideration.

**All the best**,

Seyedeh Elaheh Bagheri,

MSc in Epidemiology, Department of Epidemiology and Biostatistics, Faculty of Public Health, Tehran University of Medical Sciences, Tehran, Iran,

BSc in Nurse Anesthesia, School of Paramedicine, Guilan University of Medical Sciences, Langroud, Iran.
