# Supplementary material for: Relationship between insulin resistance surrogate markers with diabetes and dyslipidemia: A Bayesian network analysis of Korean adults
Source: PLoS One. 2025 May 8;20(5):e0323329. doi: 10.1371/journal.pone.0323329 (PMC12061414; doi:10.1371/journal.pone.0323329)
Supplement: Attachment — Submitted filename: reviewer 2_PONE-D-25-06103.docx [file pone.0323329.s003.docx]

PONE-D-25-06103

Relationship between insulin resistance surrogate markers with diabetes and dyslipidemia: A Bayesian network analysis of Korean adults

**Response to Reviewer 2 Comments**

***Thank you very much for your comments which greatly help to improve the article. We appreciate the time and effort that you dedicated to providing feedback on our manuscript. The comments are repeated in blue and responses are inserted after each comment.***

1. Uniformity in Decimal Usage: The manuscript should maintain a consistent format for decimal representation throughout. For example, please standardize the use of either one decimal place (e.g., .0) or two decimal places (e.g., .00) across the entire document.
▶ We have standardized the decimal format to two decimal places in the tables and main text. However, some figures automatically display values with three decimal places, and modifying them is not feasible due to software constraints. We hope this is acceptable, but please let us know if further adjustments are required.

2. Abbreviation Table: It is recommended to include a table of abbreviations. While abbreviations should be defined upon first use in the text, a dedicated table will provide readers with a quick reference, enhancing clarity and understanding of the terms used throughout the study.

▶ In accordance with PLOS ONE guidelines, abbreviations are defined upon their first use in the text. Given this, we believe a separate abbreviation table is unnecessary, as it would increase the document length without adding significant benefit. However, if you strongly recommend it, we are open to reconsidering.

3. Citing Figures and Tables: Ensure that figures and tables are appropriately cited within the results section. Additionally, the discussion section should include interpretations of these figures and tables to provide context and enhance the reader's understanding of the findings.
▶ We have revised the Results section to ensure appropriate citations of figures and tables, adding more explanations and interpretations where necessary. In the Discussion section, we have interpreted the results and cited the relevant figures and tables when appropriate. In the revised manuscript, additions are highlighted in red.

4. Future Research Directions: In the limitations section, please provide a brief discussion on potential future research directions that could address the identified limitations. This would offer valuable insights into how subsequent studies might build upon your findings.

▶ In the revised manuscript, we have included a discussion on potential future research directions as follows:

‘Finally, it is important to note that our Bayesian network analysis and the resulting DAG do not establish causal relationships between variables. The arrows in our DAG represent probabilistic associations and conditional dependencies learned from the data, rather than proven causal pathways [19]. While these associations provide valuable insights into the relationships between IR markers and metabolic disorders, causal inference would require different methodological approaches, such as interventional studies or causal inference frameworks with additional assumptions [52]. Therefore, our findings should be interpreted as revealing strong statistical associations rather than definitive causal mechanisms. Given that a network structure model was derived in this study, we propose exploring causal relationship inference based on this model as a potential direction for future research.’

5. Public Health Implications: The discussion section currently lacks a thorough exploration of the public health implications of your findings. It would be beneficial to elaborate on how these results could influence public health strategies and clinical practices related to diabetes and dyslipidemia management.
▶ We have added the following paragraph in Discussion section;

‘Our findings have significant clinical implications. The TyG index, centrally positioned in our network, offers a cost-effective screening tool using only routine measurements. Its robust associations with both diabetes and dyslipidemia support its use for early risk assessment, potentially improving screening efficiency and resource allocation, especially in resource-limited settings. These insights could enhance early detection and targeted interventions for cardiometabolic diseases.’

6. Connection Between Limitations and Objectives: A more explicit connection between the limitations of current methods and your study's objectives would strengthen the rationale for your research. This would help clarify why the study is necessary and how it contributes to the existing body of knowledge.
▶ We have added the following paragraph in Limiting section;

‘However, our use of the Bayesian networks offers advantages over traditional regression models by enabling unsupervised learning of probabilistic relationships and providing a visual representation through DAGs. This approach captures complex interrelationships that regression models may overlook.

Furthermore, existing methods for assessing IR and metabolic disorders are often invasive, costly, or complex, limiting their clinical utility. Our study addresses this gap by employing Bayesian networks to identify probabilistic relationships among IR markers and metabolic disorders, offering a novel and more accessible tool for risk assessment in routine clinical practice.’

7. Streamlining Redundant Information: Some sections of the manuscript contain redundant information that could be streamlined to maintain reader interest and focus. For instance, the detailed explanation of hyperglycemia mechanisms could be condensed to allow for a more direct presentation of the study's aims.
▶ We have revised the mentioned section for conciseness as follows. Please let me know if there are other areas that could benefit from further streamlining.

‘Hyperglycemia damages blood vessels by triggering inflammation, promoting lipid synthesis, and inducing oxidative stress, leading to vascular remodeling and plaque formation [3–5]. These changes increase the risk of complications across multiple organs. Accurate IR assessment is crucial for prevention and management. While the euglycemic clamp is the gold standard for measuring IR, its invasiveness, time demands, and cost limit its clinical use [6–8].’

8. Presentation of Quartile Data: The presentation of quartile data for the HOMA-IR, TyG index, and TG/HDL ratio is overly detailed. The extensive listing of percentages across quartiles may overwhelm readers and obscure the main findings. Summarizing these results in a more concise manner, potentially through visual aids or summary statistics, would improve clarity and focus.
▶ We have created a comprehensive table that clearly demonstrates the distributions of IR indices across quartiles This table allows readers to quickly identify significant patterns across all IR indices. Accordingly, we have included explanations of this table as follows;

‘Patients with diabetes showed high insulin resistance markers, with the majority falling in the highest quartile group (Q4) across all indices. Specifically, for TyG index, 52.66% were in Q4 and 25.53% in Q3. HOMA-IR values were in Q4 at 58.09%, with 21.21% in Q3. The TG/HDL ratio showed 38.28% in Q4 and 28.90% in Q3.

Among dyslipidemia patients, a similar pattern emerged but with slightly different distribution. For TyG index, 42.81% were in Q4 and 25.71% in Q3. HOMA-IR measurements showed 35.48% in Q4 and 28.10% in Q3. The TG/HDL ratio showed 44.48% in Q4 and 24.84% in Q3.’

**Table 2. Summary of IR indices across quartiles**

| **Variables** | **Levels** | **Diabetes** | | | **Dyslipidemia** | | |
| --- | --- | --- | --- | --- | --- | --- | --- |
|  |  | **No** | **Yes** | **p-value** | **No** | **Yes** | **p-value** |
|  |  | **6,882 (84.80%)** | **1,313 (15.20%)** |  | **3,916 (48.14%)** | **4,279 (51.86%)** |  |
| TyG | Q1 (≤8.22) | 2,032 (28.30%) | 93 (6.52%) | < 0.01 | 1,524 (38.11%) | 601 (12.81%) | < 0.01 |
|  | Q2 (8.22-8.66) | 1,886 (26.77%) | 209 (15.29%) |  | 1,242 (31.87%) | 853 (18.67%) |  |
|  | Q3 (8.66-9.11) | 1,688 (24.88%) | 351 (25.53%) |  | 922 (24.20%) | 1,117 (25.71%) |  |
|  | Q4 (>9.11) | 1,276 (20.05%) | 660 (52.66%) |  | 228 (5.82%) | 1,708 (42.81%) |  |
| TG/HDL | Q1 (≤1.37) | 1,991 (27.47%) | 168 (11.31%) | < 0.01 | 1,512 (37.44%) | 647 (13.48%) | < 0.01 |
|  | Q2 (1.37-2.27) | 1,811 (25.62%) | 286 (21.51%) |  | 1,295 (33.39%) | 802 (17.20%) |  |
|  | Q3 (2.27-3.83) | 1,633 (24.28%) | 383 (28.90%) |  | 959 (25.14%) | 1,057 (24.84%) |  |
|  | Q4 (>3.83) | 1,447 (22.63%) | 476 (38.28%) |  | 150 (4.03%) | 1,773 (44.48%) |  |
| HOMA-IR | Q1 (≤1.15) | 1,946 (28.08%) | 106 (7.98%) | < 0.01 | 1,365 (35.50%) | 687 (15.29%) | < 0.01 |
|  | Q2 (1.15-1.75) | 1,887 (27.10%) | 175 (12.72%) |  | 1,128 (29.00%) | 934 (21.13%) |  |
|  | Q3 (1.75-2.79) | 1,774 (25.74%) | 296 (21.21%) |  | 867 (21.77%) | 1,203 (28.10%) |  |
|  | Q4 (>2.79) | 1,275 (19.08%) | 736 (58.09%) |  | 556 (13.73%) | 1,455 (35.48%) |  |

P-values were obtained using the Rao-Scott chi-square tests. Abbreviations: TyG, triglyceride-glucose index; HOMA-IR, homeostasis model assessment of insulin resistance; TG/HDL, triglyceride-to-high-density lipoprotein cholesterol ratio; Q1, 1st quartile group; Q2, 2nd quartile group; Q3, 3rd quartile group; Q4, 4th quartile group.

▶ Moreover, we have added the following paragraph in Statistical analysis section:

‘For each IR index, participants were stratified into quartiles: Q1 (≤ first quartile), Q2 (between the first and second quartiles), Q3 (between the second and third quartiles), and Q4 (> third quartile). Distribution across quartile groups was analyzed by diabetes and dyslipidemia status, with tests for significant differences.’

9. Exclusion of Sleep Duration and Confounders: You mention the exclusion of sleep duration from the Directed Acyclic Graph (DAG) structure but do not explain how this exclusion may have impacted the results. Additionally, the mention of "potential unmeasured confounders" is too general; please specify what these confounders might be and how they could influence the study's findings.
▶ In this study, we selected a model based on high validity and effective explanation of relationships between the IR index, diabetes, dyslipidemia, and clinical variables. To ensure robustness, we performed model averaging through 1,000 bootstrap samples.

With a lower threshold (0.55–0.70), sleep duration appeared as an upper-level parent node of sociodemographic variables, but at 0.75 or higher, this connection disappeared (observed in <750 samples). The highest-level parent node directly influences its child nodes. However, if the variables forming the Markov Blanket of a given node are known, the node can be predicted without the need for the highest-level parent node (Markov blanket principle). Since the stronger threshold (0.75) yielded a more explanatory DAG, we selected a model excluding sleep duration.

While this exclusion may raise concerns, our modeling approach suggests it unlikely impacted the study’s main conclusions.

Moreover, the **sleep variable's format change** in 2021 may have affected its integration. Its **age-sensitive nature** may also be diluted in our **nationwide, diverse-age sample.**

We have revised corresponding sentences as follows:

‘Our research lacked direct IR validation against actual measurements and excluded sleep duration from the DAG structure despite its presence at lower thresholds (0.55-0.70), as it disappeared at stronger thresholds (0.75+) and showed no probabilistic influence on key variables. This exclusion likely had minimal impact, though a 2021 format change and our age-diverse sample may have contributed to this pattern.’

▶ Additionally, we have revised confounders part as follows:

Additional limitations include potential unmeasured confounders such as dietary patterns, occupational factors, and psychiatric conditions that could influence IR pathways—a constraint inherent to observational studies, limited sample size due to recent insulin measurement data unavailability, possible result variation based on hyperparameter settings, and limited generalizability beyond the Korean population.

***Thank you very much!***
